# Editorial Comment to Case of atypical femoral fractures that mimicked the typical imaging findings of prostate cancer‐induced bone metastasis

**DOI:** 10.1002/iju5.12116

**Published:** 2019-09-11

**Authors:** TP Rajeev

**Affiliations:** ^1^ Department of Urology K.S. Hegde Medical Academy NITTE (Deemed to be University) Mangalore Karnataka India

The article by Nezu *et al*. is an interesting one which highlights the increasing incidence of newer disease entities as a side effect of advancements in bone resorption inhibitor (BRI) therapy.^1^ Although medication‐related osteonecrosis of jaw (MRONJ) has been an established complication of bisphosphonates and denosumab, other osteopathological lesions are now being known to occur and the treating physician needs to be aware of it. By reporting a case of atypical femoral fracture (AFF) in a patient who had received denosumab, the authors have brought out how to tackle the clinical dilemma as well as made efforts to focus on the proper investigation tools which will be a guide for others in the future.

BRI have been in use for nearly two decades with the introduction of bisphosphonates in 2001 and the receptor activator of nuclear factor kappa B ligand inhibitor denosumab in 2010.^2^ They have changed the treatment scenario of metastatic cancer by improving the quality of life and by prolonging the life span. But Marx^3^ reported the association of osteonecrosis of jaw with bisphosphonates in 2003 and thereafter case reports of MRONJ flooded the literature. Currently there are a growing number of case reports of AFF^4^ in association with BRI treatment. Another site that can be affected is humerus. I believe that MRONJ, AFFs, and low energy fractures of the humeral shaft are “cluster diseases” which occur in association with BRI therapy. Hence patients on BRI need proper monitoring to identify and prevent such complications. Aside from that, the treating physician should also look for other potential and vulnerable sites in the skeletal system for such osteopathological lesions that have not been identified to date.

Differentiating metastatic lesions from “cluster diseases” will be challenge in clinical practice. It has been proven that metastasis has a predictable course with malignancy^5^ and knowledge of this pattern will help in its early detection. The medical practitioners and radiologists should be aware of the “cluster diseases” that can occur as a complication of BRI therapy. To our surprise, there is a significant level of ignorance about even MRONJ among medical practitioners,^6^ which needs to be addressed on a priority basis. Also, the most important diagnostic tool for detection in the future would be “clinical suspicion” than any investigative modality, as most patients have terminal malignancies and hence subjecting them to aggressive medical testing should be avoided.

## Conflict of interest

The author declares no conflict of interest.
